# Ventricular Free Wall Rupture: Ten Year Survival After Surgical Repair

**DOI:** 10.2174/1874192400802010001

**Published:** 2008-01-22

**Authors:** Richard A Leff, Irwin Hoffman

**Affiliations:** Lovelace Medical Center, Division of Cardiology, 5400 Gibson Blvd, SE, Albuquerque, NM 87108, USA

**Keywords:** Ventricular rupture, Long term survival, Surgical repair

## Abstract

Ventricular free wall rupture is a devastating complication of acute myocardial infarction. It occurs in 15-25% of fatal cases. However, the overall incidence in acute MI cases is about 2%. [1] Clinical markers suggesting free wall rupture include pulseless electrical activity in a first MI, and pericardial tamponade. Subacute rupture takes hours or days to develop, and is suggested clinically by pericardial pain, transient hypotension, nausea, restlessness and agitation. [2, 3] When the diagnosis is established by pericardiocentesis or echocardiography, surgical patch repairs are possible, using standard or even sutureless technique. [4] The long term course of survivors of free wall rupture repair has not been extensively reported. There are scattered reports in the literature of survival up to eight years. [5, 3] We report herein a case of a status freewall rupture from an inferior-posterior wall myocardial infarction with survival of ten years after surgical repair. We believe this to be the longest survival thus far reported in the literature.

## Case report

1F.R., a 58 year old Hispanic male, DOB 1/10/1937, sustained an acute inferior-posterior myocardial infarction on 9/25/1996. Coronary angiography demonstrated a totally occluded circumflex coronary artery filling *via *collaterals, with patent left anterior descending and right coronary vessels. Echocardiography showed inferior-posterior akinesis, inferior wall aneurysm, and an ejection fraction about 40%.

Because of recurrent ventricular tachycardia with syncope, he received a permanent defibrillator implant about two weeks after the acute MI. One week later he was readmitted with syncope and physical signs of cardiac tamponade. Emergent cardiac catheterization and ventriculography indicated ventricular rupture with extravasation into the pericardium. At surgery this diagnosis was confirmed and the ventricular defect was closed with a Teflon patch. No coronary bypass grafting was done. Postoperative echocardiography revealed the patch and a small residual pericardial effusion. Ten years after surgical repair, the patient is asymptomatic on a secondary prevention regimen of an aspirin, ace inhibitor, a statin, and a beta-blocker. His favorable course illustrates that myocardial rupture after acute MI, although most often rapidly fatal, can occasionally be diagnosed in time for a surgical repair to be performed. In this case, the patient has survived and has been well for ten years, despite the additional co-morbidity of recurrent ventricular tachycardia requiring an implantable defibrillator. We believe that the long term favorable course in our case is attributable to preserved ventricular function. As in the case of infarction complicated by ventricular septal or papillary muscle rupture, survival after repair of the mechanical complication depends on adequate ventricular function. In our patient, with a current ejection fraction of 50 %, he has not only survived for ten years- but is asymptomatic and fully active.

2,3

## Figures and Tables

**Fig. (1):**
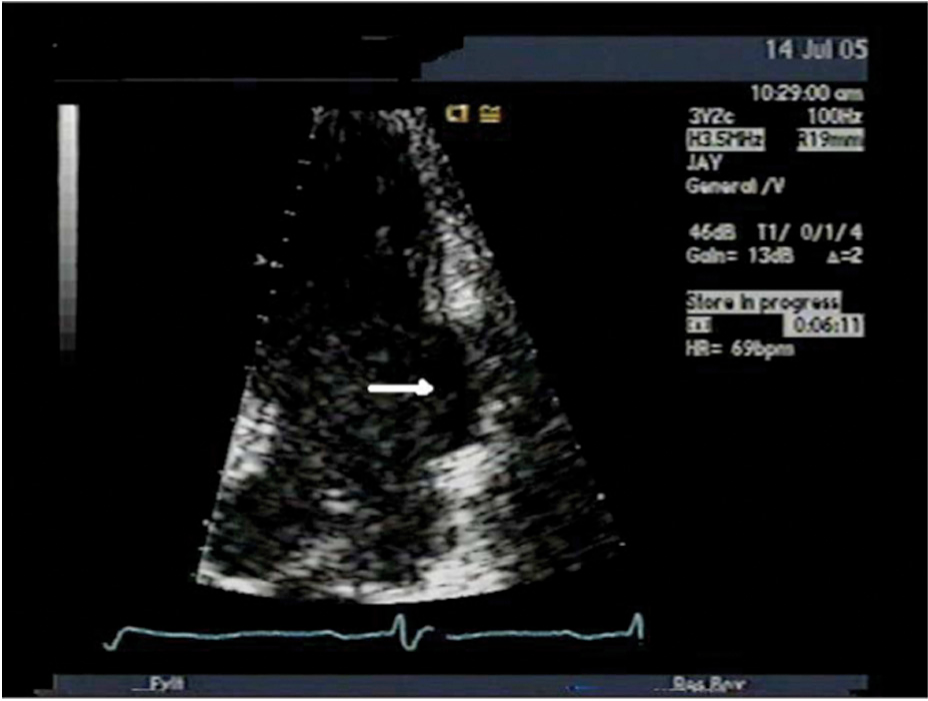
Transthoracic echocardiogram done 10 years after patch repair of the subacute ventricular rupture (arrow).

**Fig. (2):**
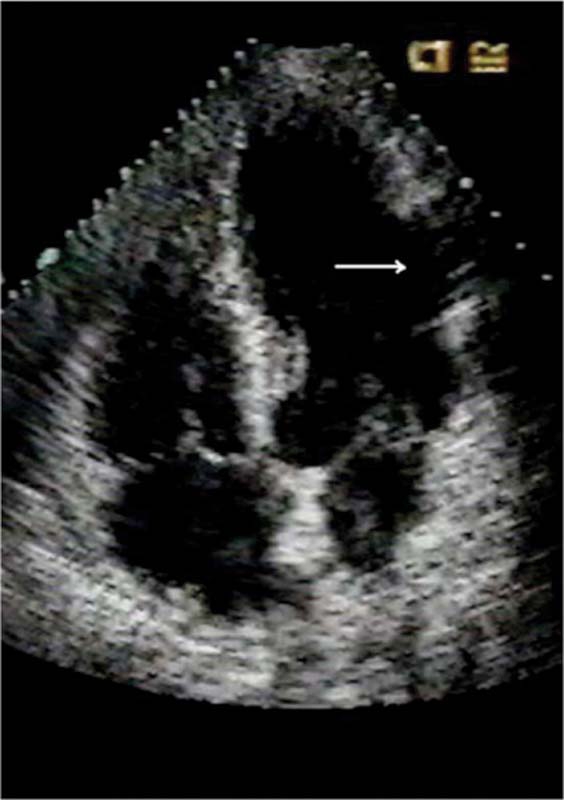
Transthoracic echocardiogram done 10 years after patch repair of the subacute ventricular rupture (arrow).

**Fig. (3):**
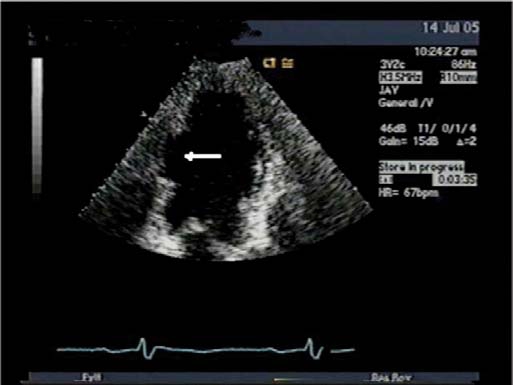
Transthoracic echocardiogram done 10 years after patch repair of the subacute ventricular rupture (arrow).
